# Mammary neoplasms in female dogs: Clinical, diagnostic and therapeutic aspects

**DOI:** 10.17221/4/2024-VETMED

**Published:** 2024-04-26

**Authors:** Janaina Reato Rueda, Camila Dias Porto, Rodrigo Prevedello Franco, Isabela Bazzo da Costa, Lais Melicio Cintra Bueno, Raul Jose Silva Girio, Fabio Fernando Ribeiro Manhoso, Patricia Cincotto dos Santos Bueno, Claudia Sampaio Fonseca Repetti

**Affiliations:** Department of Veterinary Science, University of Marilia – UNIMAR, Marilia/SP, Brazil

**Keywords:** dogs, mammary tumour, mammary lump, manual, mammary carcinoma

## Abstract

With the increase in the life expectancy of domestic animals and their increasingly affectionate relationship with their owners, it is possible to observe an increase in cases of neoplasms in these animals. Mammary neoplasia mainly affects older females who have not been castrated, due to hormonal dependence for the development of the tumour. The main form of treatment is surgery. This study aims to carry out an updated review on mammary neoplasms in female dogs covering the anatomy, physiology, prevalence, causes, diagnoses, treatments, prevention and prognosis, based on scientific articles by renowned researchers.

## INTRODUCTION

The occurrence of neoplasms in domestic animals, especially in dogs, has increased due to the greater longevity of these animals. This may be due to the use of balanced diets, vaccination schedules, advanced diagnostic techniques, and increasingly specific and effective therapeutic protocols (Reis et al. 2010; Ribas et al. 2012; Biondi et al. 2014). This fact has allowed major advances in veterinary oncology, which stands out in the search for improvements in the prevention, diagnosis and treatment of neoplasms (Beauvais et al. 2012; Nagata et al. 2014).

Research involving mammary neoplasms in canine and feline females has gained importance, on the one hand, due to the similarities, in some aspects, to women and, on the other hand, due to the frequency with which they appear in companion animal clinics (Silva et al. 2004). Complete knowledge about the care and diagnosis of breast cancer in women is extremely important for public health, motivating studies on prevention and early diagnoses that seek to reduce morbidity and mortality related to this neoplasm (Humphrey et al. 2002). Also in veterinary medicine, there is great interest on the part of researchers, mainly due to the high casuistry of oncological care, often with late diagnoses, which compromises the success of treatment and reduces the survival rate of patients (Silva et al. 2004; Cavalcante and Cassali 2006; Andrade et al. 2010; Estrela-Lima 2010).

Mammary tumours are the most common neoplasms in veterinary oncology services in Brazil, representing more than 50% of the tumours diagnosed in dogs (Ribas et al. 2012; Biondi et al. 2014; Collivignarelli et al. 2021), the majority of which are malignant (Biondi et al. 2014; Valdivia et al. 2021). Metastases and recurrences develop in about 35–70% of bitches after excision (Collivignarelli et al. 2021).

## LITERATURE REVIEW

### Surgical anatomy

The female dog has two mammary chains, with five glands each, arranged in parallel from the thoracic region to the inguinal region, named cranial thoracic (M1) and caudal (M2), cranial abdominal (M3) and caudal (M4) and inguinal (M5). Macroscopically, the mammary gland has bilaterally symmetrical and parallel glandular units in the ventral midline (Dyce et al. 2010), and, microscopically, it is a modified exocrine, tubuloalveolar sweat gland, responsible for the production and secretion of milk, specific to mammals (Getty 2008; Reese et al. 2011).

Regarding the blood supply to the mammary glands in bitches, the cranial and caudal thoracic mammary glands receive arterial blood from the internal thoracic, intercostal and lateral thoracic arteries; the cranial abdominals are irrigated by the cranial superficial epigastric artery and the caudal and inguinal abdominals are mainly irrigated by the caudal superficial epigastric artery (Dyce et al. 2010; Reese et al. 2011). Venous drainage of glands acts like arterial irrigation, however, small veins may cross the midline between the right and left glands (Reese et al. 2011).

The lymphatic drainage of the mammary chains in bitches is complex. In general, the cranial and caudal thoracic mammary glands drain into the ipsilateral superficial axillary and cervical lymph nodes (Lana et al. 2007; Dyce et al. 2010). The cranial abdominal breast drains mainly into the axillary lymph node, but simultaneously drains into the inguinal lymph node, both ipsilateral. The caudal and inguinal abdominal breasts drain into the ipsilateral superficial inguinal lymph node (Cassali et al. 2014). Drainage to the contralateral lymph node has already been described and may occur through lymphangiogenesis induced by the neoplasia (Sorenmo et al. 2011).

According to Sorenmo et al. (2011) some differences can be observed in the drainage of mammary glands without neoplasms when compared to neoplastic mammary glands (Table 1).

**Table 1:** Normal and neoplastic lymphatic drainage of mammary gland tumours in bitches

Mammary gland	Normal lymphatic drainage	Neoplastic lymphatic drainage
M1	axillary lymph node	axillary and sternal lymph node
M2	axillary lymph node	axillary and sternal lymph node
M3	axillary and superficial inguinal lymph node	axillary, superficial inguinal and medial iliac lymph nodes
M4	superficial inguinal lymph node	superficial inguinal and axillary lymph node
M5	superficial inguinal lymph node	superficial inguinal, popliteal and medial lymph nodes on the thigh

Source: Adapted from Sorenmo et al. (2011)

The lymphatic system is the main route for the generation of metastases from malignant mammary tumours in dogs (Queiroga and Lopes 2002), therefore, the clinician and surgeon require knowledge regarding mammary lymphatic drainage for the adequate surgical excision, as well as for prognostic determination (Cassali et al. 2014).

Inspection of the regional lymph node should be part of the routine evaluation of mammary neoplasms in bitches, as the presence of metastases has an impact on the neoplastic staging, survival and treatment to be recommended (Cassali et al. 2014).

### Aetiology and predisposing factors

The aetiology of breast tumours may be related to dietary, genetic, environmental and hormonal factors (Henderson and Feigelson 2000; Silva et al. 2004; Uva et al. 2009; Andrade et al. 2010; Ribas et al. 2012; Toribio et al. 2012), with especial emphasis on this last factor, hormonal.

Despite the epidemiological importance of breast tumours in bitches in Brazil, medical records of the care for these animals are often incomplete (Biondi et al. 2014), missing important information about the reproductive life such as castration, occurrence of illnesses reproductive disorders (pseudocyesis, abortion and others) and use of contraceptives which are often related to the aetiology of these tumours in bitches (Ribas et al. 2012; Toribio et al. 2012).

In both normal and neoplastic breasts, tissue growth is stimulated by hormones, oestrogen, progesterone and prolactin, and growth factors (Misdorp 2002; Sorenmo et al. 2013; Cassali et al. 2014; de Araujo et al. 2015).

Oestrogen plays an important role in oncogenesis as it stimulates the mitotic activity of the mammary epithelium, which increases the risk of neoplasms in this organ. The expression of the oestrogen receptor (ER) is undoubtedly an important biomarker in human breast cancer, as its presence determines the sensitivity index to hormonal treatment (Buitrago et al. 2011). In bitches, the proportion of benign mammary tumours that present an increase in ERs and progesterone receptors (PRs) is high, while carcinomas present a lower concentration of these receptors (Horta et al. 2012).

Progesterone acts to suppress the activity of the myometrium, stimulates the growth of endometrial glands and promotes the development of breast alveolar tissue (Martins and Lopes 2005). When associated with oestrogen, it plays an important role in the development of breast neoplasms, due to the proliferative factor (Misdorp 2002; Queiroga et al. 2010). When associated with prolactin, it increases the number of oestrogen receptors, facilitating the mitotic action of this hormone (Misdorp 2002).

Given the above, one of the main risk factors for tumour development are sex hormones (Beauvais et al. 2012). Furthermore, the occurrence of pseudocyesis and the use of progestins as contraception may contribute to the formation of these tumours. When administering exogenous progesterone to dogs or cats, there is stimulation of the growth hormone synthesis in the mammary gland, lobuloalveolar proliferation and consequent hyperplasia of the myoepithelial and secretory elements, inducing the formation of benign nodules in young animals (Rutteman et al. 2001). Breast tissues can undergo malignant metaplasia when constantly exposed to progesterone (Misdorp 2002).

In pseudocyesis, the non-pregnant female presents an increase in serum levels and/or sensitivity to prolactin, associated with a faster than normal decrease in progesterone. In bitches that present recurrent pseudocyesis, high concentrations of prolactin cause milk retention and the possible formation of mammary neoplasms (Martins and Lopes 2005).

Some studies show a possible relationship between obesity and the development of mammary neoplasms in bitches (Cleary et al. 2010; Lim et al. 2015; Chandler et al. 2017). Obesity in the development of these neoplasms may be related to the actions of leptin and adiponectin proteins (Cleary et al. 2010). Leptin inhibits apoptosis and stimulates cell proliferation while adiponectin reduces cell proliferation and promotes apoptosis. These substances produced by the adipose tissue undergo changes in concentrations according to the body weight, so when the weight and body mass index increase, the serum concentrations of leptin increase and adiponectin decrease (Cleary et al. 2010; Lim et al. 2015).

Obesity may also be associated with a decrease in the serum concentration of sex hormones bound to the globulin, resulting in an increase in the serum concentrations of free oestrogen. Furthermore, adipose tissue can be a source of increased oestrogen production via aromatase mediated androgen conversion (De Nardi et al. 2016).

In a study carried out by Sirivaidyapong (2003), 91.3% of bitches that received snacks or were fed a homemade diet mixed or not with food, had mammary tumours. Homemade food, rich in meat and animal fat, causes, in addition to obesity, an increase in the emergence of mammary neoplasms, with these factors having a greater role in promoting carcinogenesis (Cotran et al. 2000; Sirivaidyapong 2003).

There are few studies correlating the type of diet with carcinogenesis in animals, however, there are some reports in humans, and the diet can cause 20% to 30% of all cancers in humans in economically developed countries. Some research reveals that animals that received homemade food, mainly meat, especially pork¸ increased the development of dysplasia and mammary tumours. There is a higher prevalence of mammary neoplasia in bitches that are obese and that received a diet rich in red meat (De Nardi et al. 2016).

### Diagnosis

#### CLINICAL APPROACH AND STAGING

The majority of bitches with mammary neoplasms are clinically healthy at the time of diagnosis and the nodules can be identified by owners or veterinarians during routine clinical examination (Cassali et al. 2011). However, according to Oliveira et al. (2021), approximately 25% to 50% of dogs with malignant mammary tumours already have metastases at the time of diagnosis.

The early diagnosis of mammary gland neoplasms is essential, through a complete physical evaluation, laboratory tests, imaging diagnoses and the microscopic evaluation of the nodule, which will be fundamental for establishing adequate treatment, essential in preventing recurrence or metastasis (Novosad 2003).

Information regarding the time of onset and progression of the lesion, previous treatment and reproductive history is important (Cassali et al. 2014). After the anamnesis and complete physical examination to determine the general condition (Cassali et al. 2014), the female must undergo a specific physical examination of the mammary gland, which consists of palpation of the mammary chains and regional lymph nodes to identify nodules or changes in the size, shape or consistency, macroscopically defining the lesions (Giuliano et al. 2011; Cassali et al. 2014; Brissot and Edery 2017).

After palpation of the mammary gland and lymph nodes, a fine needle cytopathological examination (FCAA) of the nodules and lymph nodes should be used as a complementary part of the diagnosis (De Nardi et al. 2016). According to Lana et al. (2007), there is no predisposition regarding the mammary chains affected in the neoplastic process, however, some authors reported that approximately 60% of neoplasms occur in the last two pairs of glands, that is, the caudal and inguinal abdominals, as they have a greater volume of glandular tissue (Misdorp 2002; Goldschmidt et al. 2017).

Regarding the morphology, they can appear as small or large nodules, plaques, adherent or mobile, single or multiple, ulcerated, or circumscribed, depending on the biological behaviour of the tumour (Misdorp 2002; Sorenmo et al. 2011; Goldschmidt et al. 2017).

Nodules larger than five centimetres, fast-growing, adherent, with large areas of ulceration and with metastases to the lymph nodes are associated with the worst prognosis (Ferreira et al. 2009; Estrela-Lima 2010; Beserra et al. 2016). Breasts with multiple nodules can present different histological types, both benign and malignant, these aspects must be taken into consideration, as they influence the prognosis (Cassali et al. 2014).

Regarding the fact that there is no breed predisposition (Dore et al. 2003), however, some studies demonstrate a higher incidence in Cocker Spaniel, Dachshund, English Setter, Pointer, Fox Terrier, Boxer and Beagle bitches (Dore et al. 2003). However, with the breed, it is important to consider that breed predisposition is not a determining factor, as there is a large percentage of dogs of no defined breed being affected by mammary neoplasia, concluding that it is complex to establish a standard of predisposed breeds (Misdorp 2002; Zatloukal et al. 2005).

Clinical staging of bitches with mammary neoplasms must be carried out so that planning can be carried out regarding treatment options and the prognosis can be determined (Batschinski and Tedardi 2016). The World Health Organization (WHO) proposed the TNM system whose variables include the assessment of the primary tumour (T), involvement of the regional lymph nodes (N) (axillary and superficial inguinal) and identification of distant metastases (M) (Rutteman et al. 2001; Batschinski and Tedardi 2016; De Nardi et al. 2016; Cassali et al. 2020). This classification system defines five clinical stages according to the tumour progression (Table 2).

**Table 2:** Clinical staging for bitches with mammary neoplasms according to the TNM system

**T – Primary tumour**
T1: < 3 cm diameter
T2: 3–5 cm diameter
T3: > 5 cm diameter

**N – Regional lymph nodes**
N0: absence of metastasis (cytology or histology)
N1: presence of metastasis (cytology or histology)

**M – Distant metastasis**
M0: absence of metastasis
M1: distant metastasis

**Stages**
I: T1N0M0
II: T2N0M0
II: T2N0M0
III: T3N0M0
IV: Any TN1M0
V: Any T any N M1

Source: Adapted from Cassali et al. (2011)

Patients with stage IV or V, as well as those with nodules larger than five centimetres (T3), have a negative impact on the prognosis, with shorter survival times than others (Cassali et al. 2020). According to Nunes et al. (2018), dogs in clinical stage IV had a median survival time of 331 days compared to 1 149 days for dogs in stage I.

Palpation of the superficial axillary and inguinal lymph nodes should be performed in search of changes. It is important to highlight that the absence of inflammation and lymph node enlargement does not exclude neoplastic involvement of the lymph nodes, and cytology should be performed on the regional lymph nodes and a histopathological examination should be performed when a mastectomy is performed, being considered important factors in the prognosis having a great impact on the survival of dogs (Nunes et al. 2018; Cassali et al. 2020).

According to Oliveira Filho et al. (2010), more than 25% of cases of mammary cancer present with lymph node metastasis. The distribution of these metastases is determined according to the blood and lymphatic drainage of the primary tumour, with the preferred sites for metastasis being regional lymph nodes and mainly the lungs (De Nardi et al. 2013; Nunes et al. 2018), justified due to the intense flow of blood that passes through this organ, in which a network of capillaries slows circulation and acts as a filter for aggregates of tumour cells, which lodge in the vascular tree, inserting pseudopods between the endothelial cells and migrating to the lung parenchyma (Cotram et al. 2000).

Imaging exams play a fundamental role in helping both the initial diagnosis and the therapeutic response in oncological cases (Oliveira et al. 2021); The radiographic examination is extremely important for the staging of animals, as one of the sites most affected by distant metastasis is the lung. Therefore, all bitches with mammary tumours need to undergo radiography in three views (right and left lateral and ventrodorsal). However, pulmonary micrometastases (nodules between 0.2 mm and 2 mm) are not visualised using chest radiography. Therefore, if metastases are not identified on the chest X-ray examination, the presence of metastases cannot be ruled out and tomography examinations may be indicated (Dobson and Lascelles 2011).

An abdominal ultrasound is also indicated for metastasis research, as it allows for the evaluation of the echotexture of the tumour process and its invasiveness, which will help in planning the surgical procedure (Nunes et al. 2018). Computed tomography allows the identification of metastatic nodules smaller than 6 mm, being considered the gold standard for researching metastasis in both the thoracic and abdominal cavities (Cassali et al. 2014).

The investigation of distant metastasis is essential to determine the clinical staging and therapeutic plan. Dogs with distant organs infiltrated by tumour cells may not benefit from surgery (Nunes et al. 2018).

#### MICROSCOPIC EVALUATION

The microscopic characteristics of the tumour associated with clinical staging are important tools for determining the prognosis and defining the best therapeutic strategy (Cassali et al. 2020).

#### HISTOLOGIC EVALUATION

A histopathological evaluation, commonly performed after surgery, is recommended for all cases and, in addition to the primary tumour, all mammary glands and regional lymph nodes must be evaluated (Cassali et al. 2020). In case of multiple nodules, the histopathological diagnosis should be performed on all of them, which will be useful in determining the prognosis and additional treatment options (Litterine-Kaufman et al. 2019).

Despite the existence of different tumour classification methods and the absence of uniform criteria to differentiate types of neoplasms, in Brazil, Cassali et al. (2020) published an updated histological classification (Table 3).

**Table 3:** Histological classification of canine mammary tumours

**Histological classification of canine mammary tumours**	
**Non-neoplastic epithelial lesions**	
Ductal hyperplasia	Nodular hyperplasia
Adenosis	Ductal ectasia
Columnar cell injuries:	
- Columnar cell changes	- Columnar cell hyperplasia
- Atypical columnar cell lesion	
	
**Benign tumours**	
Adenoma	Adenomyoepithelioma
Myoepithelioma	Basaloid adenoma
Fibroadenoma	Benign mixed tumour
Ductal papilloma	Phyllodes tumour
	
**Malignant tumours**	
- Carcinomas	
Carcinoma *in situ*: ductal carcinoma *in situ*, lobular carcinoma in situ	
Carcinoma in mixed tumour	
Papillary carcinoma (invasive and non-invasive)	
Tubular carcinoma	Solid carcinoma
Basaloid carcinoma	Cibriform carcinoma
- Special types of carcinomas	
Micropapillary carcinoma	Pleomorphic lobular carcinoma
Secretory carcinoma	Mucinous carcinoma
Lipid-rich carcinoma	Glycogen-rich carcinoma
Squamous cell carcinoma	Fusiform cell carcinoma
Carcinoma with sebaceous differentiation	
	
- Myoepithelial neoplasms	
Malignant adenomyoepi thelioma	Malignant myoepithelioma
	
- Sarcomas	
Fibrosarcoma	Osteosarcoma
Carcinosarcoma	Sarcoma in mixed tumour
	
- Other sarcomas	
Condrosarcoma, Liposar coma, Hemangiosarcoma, Phyllodes sarcoma	

Source: Cassali et al. (2020)

The histopathological classification of mammary neoplasms in bitches is an important tool to indicate the biological behaviour of the neoplasm, however, it is controversial among pathologists due to the histogenetic complexity. The most used classifications are those described by Moulton (1990), from the World Health Organization (WHO) and later Goldschmidt et al. (2011), with some divergences between them. Histological types such as solid carcinoma, micropapillary carcinoma, carcinosarcomas and sarcomas are associated with an unfavourable prognosis with shorter survival time and greater risk of developing metastasis (Horta et al. 2012; Cassali et al. 2014; Nunes et al. 2014; de Oliveira Gamba et al. 2017; Nunes et al. 2019).

#### IMMUNOHISTOCHEMISTRY

The immunohistochemistry technique is used in veterinary medicine to identify various markers related to the biological behaviour of tumours to determine prognostic and predictive factors for neoplasms (Ramos-Vara et al. 2008). According to Cassali et al. (2020), the immunohistochemical panel for canine and feline mammary carcinomas should be composed of the expressions of the oestrogen receptor (ER), progesterone receptor (PR), Ki-67 and COX-2.

The Ki-67 proliferation index is a cell cycle-related marker widely used in canine neoplasms (Kaszak et al. 2018). This marker estimates the cellular proliferation potential of breast neoplasms and is related to the prognosis of these tumours. In general, tumours with higher proliferative rates exhibit more aggressive behaviour with an increased risk of metastasis (Nowak et al. 2016; Kaszak et al. 2018).

Ki-67 is a nuclear protein, non-histone, detected in cells during all the active phases of the cell cycle (G1, S, G2 and M) that disappears rapidly after mitosis. Ki-67 is considered the main prognostic marker of the cell proliferation index in canine mammary neoplasms (Cassali et al. 2014).

According to the latest Brazilian consensus, the proliferation index should be determined by Ki-67 nuclear staining evaluated in at least 1 000 neoplastic cells in high power fields (400×), also considering the size of the microscopic field and the suggested cut off point is ≥ 20% (Cassali et al. 2020).

COX-2 plays an important role in several neoplasms contributing to tumour development and angiogenesis (Lavalle et al. 2009; Carvalho et al. 2017). It participates in the metabolism of arachidonic acid, generating prostaglandins that are responsible for cell proliferation, apoptosis, modulation of the immune system and angiogenesis. Increased expression of COX-2 in canine mammary neoplasms has been associated with aggressive characteristics, such as histological grades, the mitotic index and lymph node metastasis and, consequently, a worse prognosis (Queiroga et al. 2010; Guimaraes et al. 2014; Milanta et al. 2016).

Lavalle et al. (2009) observed that the high expression of COX-2 in canine mammary neoplasms correlates with a shorter survival time. Similarly, Queiroga et al. (2010) observed that the higher the expression of COX-2 in canine mammary neoplasms, the greater the chance of lymph node and distant metastasis formation, and consequently, a shorter survival time.

Oestrogen and progesterone, in addition to being essential for the normal development of breast tissue, also influence tumour growth, since the majority of mammary neoplasms express ER and/or PR (Kim et al. 2014). Animals with mammary tumours that express ER and PR, or only ER, have a better prognosis when compared to those that express only PR, as positive ERs correlate with well-differentiated tumours (Kim et al. 2014; Mohr et al. 2016). A decreased ER/PR ratio in mammary carcinomas has been associated with decreased cellular differentiation and disease progression (Kim et al. 2014).

### Sentinel lymph node

The importance of evaluating regional lymph nodes to quantify the metastatic spread of neoplasia is well recognised in veterinary medicine, however, harvesting the correct lymph node may not be possible without mapping the sentinel lymph node (SLN) (Beer et al. 2023).

The SLN is defined as the first lymph node to drain a neoplasm, and, for this reason, it should be the first site to receive tumour cells if lymphatic dissemination occurs (Paz et al. 2001; Tuohy et al. 2009; Beer et al. 2023).

Studies show that lymph nodes corresponding to regions that have neoplasia, even without changes in size and consistency, may already have metastasis (Beserra et al. 2016).

The difficulty of lymph node resection, especially in the axillary chain, can lead to serious errors in the prognosis and the identification of metastatic focus (Matos et al. 2012). Therefore, lymph node staging techniques in bitches need to be considered, since the assessment of regional lymph nodes becomes essential for the prognosis and therapeutic approaches (Cassali et al. 2014).

The use of dyes (patent blue and methylene blue) applied to peri-neoplastic tissue has been used to identify the SLN, as they facilitate the identification of lymph nodes, in addition to minimising surgical incisions, especially when evaluating the axillary lymph node (Sorenmo et al. 2013).

The administration of patent blue dye (2 mg/kg), intradermally peritumoral, helps identify the lymphatic flow and indicates the correct SLN (Pinheiro et al. 2003; Cassali et al. 2020). For such administration, the nodule must be divided into four quadrants, and the dye distributed equally in each of them, in the intradermal region. Five to ten minutes after applying the dye, it is now possible to identify the local lymphatic drainage and the corresponding regional lymph node, through the skin or below it, after the incision (Sorenmo et al. 2013; Worley 2014; Cassali et al. 2020).

The dose of methylene blue dye used by the team of Maues et al. (2016), in bitches with mammary neoplasms, to identify the axillary SLN, was 0.5 ml for a bitch weighing up to 15 kg and 1.0 ml for those weighing more than 15 kg, in two or more applications, in the cranial thoracic breast of the mammary chain affected by the neoplasm.

According to Beserra et al. (2016), the peritumoral intradermal application of patent blue resulted in 100% specificity and 89.5% sensitivity in identifying the ipsilateral axillary lymph node in bitches with mammary neoplasms.

In a study conducted by Maues et al. (2016), 2% methylene blue administered intradermally to mammary tumours located in the cranial thoracic gland was 76.27% effective for identifying the axillary lymph node. These authors cited the advantages of this dye as being low cost, easy to access and simple to apply.

Other non-colorimetric methods for marking the SLN include indirect lymphography with iodinated oil followed by radiography (Brissot and Edery 2017), indirect computed tomographic lymphography with iopamidol (Majeski et al. 2017) or with iohexol (Rossi et al. 2018), indirect assessment using M-mode ultrasound and elastography (Silva et al. 2004; Belota et al. 2019), in addition to lymphoscintigraphy with technetium 99 (Tc-99) (Beer et al. 2023).

This last method, lymphoscintigraphy with Tc-99, is considered the standard technique for detecting SLN in human oncology (Pereira et al. 2008), but has the disadvantage of the investment costs in facilities for handling radioactive materials, in addition to the operating costs for each procedure. Such factors make its use difficult in routine veterinary oncology, and it is therefore replaced by colorimetric markers.

Buitrago et al. (2011) described that around 70% of patients with axillary lymph node involvement will develop recurrence within 10 years, compared to 20% to 30% of patients with a negative node. Identification of these lymph nodes is a challenge for staging due to the inability to visualise, which results in failures that can lead to inadequate treatment, recurrence and distant metastasis.

Paulinelli et al. (2017) considers that this approach regarding SLN research has prognostic and therapeutic value, as the identification of neoplastic cells in the lymph node is related to neoplastic aggressiveness and potential for distant metastasis, respectively, enabling the reduction of regional and distant recurrence. For Maues et al. (2016) and Collivignarelli et al. (2021), the detection of SLN is of great importance for the surgeon, not only for the most appropriate surgical excision, but also for determining an accurate surgical prognosis, and should be incorporated into the surgical routine.

### Therapy

Surgical excision is the primary treatment indicated in the control of breast neoplasms in which the objective is to remove the tumour(s) with clean margins and, depending on the case, prevent the development of new nodules in the remaining glands (Monteiro et al. 2011; Horta et al. 2014; Horta et al. 2015; Sorenmo et al. 2020). This technique can be curative in dogs without lymphatic involvement and distant metastasis or with less aggressive histological types (Horta et al. 2014; Sorenmo et al. 2020).

However, the surgery for dogs with distant metastases detected before surgery will not prolong the dog’s survival time, but may increase the quality of life of patients with ulcerated and/or painful lesions (Cassali et al. 2020).

Surgical techniques include mastectomy, which can be simple, regional, complete unilateral and complete bilateral, depending on the involvement and severity of the neoplasm. Simple mastectomy aims to remove only the affected mammary, while regional mastectomy removes mammary glands with the same lymphatic drainage. Complete mastectomy aims to remove a unilateral or bilateral chain, whether only some glands are affected or all, this technique also has a prophylactic nature, since neoplastic cells are not seen macroscopically, and can be implanted in breasts that are apparently not affected. by neoplasia (Monteiro et al. 2011).

Experts in the field have not yet reached a consensus on which technique offers better local control and reduces the risk of metastases and tumour recurrence, requiring more research (Cassali et al. 2020).

Some oncologists advocate radical procedures, considering the possibility of developing new tumours in the remaining mammary glands. Additionally, small nodules may be associated with aggressive biological behaviour. Unilateral or bilateral mastectomy, even providing greater local control of the tumour, is associated with a greater risk of surgical complications due to the aggressiveness of the technique and is not always related to a longer survival time (Horta et al. 2014; Horta et al. 2015).

Regional mastectomy assumes that some mammary glands share the same lymphatic and venous drainage and must be removed simultaneously in the bloc along with their respective superficial regional lymph nodes (Monteiro et al. 2011; Cassali et al. 2020). This technique is indicated in cases of neoplasms smaller than 3 cm (T1), slow growing, not adhered to adjacent tissues, and without signs of inflammation (Cassali et al. 2020).

Unilateral mastectomy involves the removal of all glands from a mammary chain, associated with the ipsilateral superficial regional lymph nodes (axillary and inguinal), and is indicated for multiple nodules, lesions located in M3 and tumours with poor clinical prognosis factors factors of poor clinical prognosis, such as growth rapid and/or presence of lesions larger than 3 cm (T2 and T3) (Sorenmo et al. 2020). If the patient presents nodules in both breast chains, their removal is recommended in two surgical procedures, with an interval of four to six weeks between them (Cassali et al. 2020).

For patients in clinical stage II to V, a unilateral or bilateral mastectomy may be indicated; and stage I bitches can benefit from a regional mastectomy, always considering the lymphatic drainage of the mammary chain (Cassali et al. 2020).

Table 4 shows some guidelines for determining the surgical technique for mastectomy according to Cassali et al. (2020).

**Table 4:** Guidelines for determining the surgical technique and extent for single canine mammary tumours, depending on the location

Tumour location	Tumour size	Surgery
M1*	< 3 cm (T1)	regional mastectomy (M1 – M2 and axillary lymph node)
	> 3 cm (T2 or T3)	unilateral mastectomy
M2*	< 3 cm (T1)	regional mastectomy (M1 – M2 – M3 and axillary lymph node)
	> 3 cm (T2 or T3)	unilateral mastectomy
M3*	any size (T1, T2 or T3)	unilateral mastectomy
M4*	< 3 cm (T1)	regional mastectomy (M3 – M4 – M5 and inguinal lymph node)
	> 3 cm (T2 or T3)	unilateral mastectomy
M5*	< 3 cm (T1)	regional mastectomy (M4 – M5 and inguinal lymph node)
	> 3 cm (T2 or T3)	unilateral mastectomy

*Tumours associated with other prognostic factors should undergo unilateral mastectomy (Cassali et al. 2020)

A total bilateral mastectomy consists of the simultaneous removal of the two mammary chains together with their bilateral superficial lymph nodes (axillary and inguinal), being indicated for dogs with a flat chest and elastic skin in which there is little compromise in surgical synthesis, but those animals with a deep chest will require reconstructive surgical techniques (Bartels et al. 1978). According to Cassali et al. (2020), this technique causes extensive tissue damage and should be avoided, except in cases of neoplastic invasion in the contralateral mammary chain.

The recommended surgical margin for breast tumours is 1–2 cm of healthy tissue, which may involve the adjacent muscular tissue in the deep plane (pectoral muscles, abdominal obliques or rectus abdominis), in case of tumour adherence. To plan adjuvant therapies, it is recommended to analyse the surgical margins, which can be done with nankin ink. If there are neoplastic cells in the stained area, the sample is considered to have “compromised margins” (Papazoglou et al. 2014).

When undergoing the surgical mastectomy procedure, the inguinal lymph node, due to its anatomical position, is removed next to the corresponding mammary gland. However, the axillary lymph node is only removed when there is a change in its palpation. This occurs, in part, due to the difficulty of locating it (Matos et al. 2012; Cassali et al. 2014). Studies carried out by Bianchi et al. (2018) and Collivignarelli et al. (2021) demonstrated that axillary lymph node removal should be included as a routine technique to allow better staging of mammary neoplasms in bitches.

An ovariohysterectomy can be performed at the time of mastectomy, but studies indicate that not all bitches will benefit from an increased survival time or recurrence of the disease. Those bitches who have ER-positive tumours, increased the serum oestrogen or reproductive changes unrelated to the mammary tumour may benefit from this procedure (Kristiansen et al. 2016). It should be considered that an ovariohysterectomy can lead to increased surgical trauma when performed together with a mastectomy, especially in patients who have comorbidities or an advanced stage of the disease (Cassali et al. 2020).

A palliative mastectomy aims to promote quality of life and pain control and may be indicated in patients with distant metastases and ulcerated tumours. In these cases, surgery will not influence the survival rate, promoting only the local control of neoplastic dissemination (Boston and Henderson 2014).

Although surgery is the treatment of choice, chemotherapy can help prevent recurrence and metastasis formation (Novosad 2003), and is also indicated in cases of inoperable tumours and when the patient already has distant metastases (Karayannopoulou and Lafioniatis 2016).

Bitches diagnosed with micropapillary carcinoma, solid carcinoma, carcinosarcoma and pleomorphic lobular carcinoma are at an increased risk of metastases and, in these cases, adjuvant chemotherapy is recommended, regardless of the clinical stage.

Patients with clinical stages IV and V, who have metastasis in the lymph nodes or lungs, will always benefit from chemotherapy (Cassali et al. 2011; Cassali et al. 2020).

Chemotherapy protocols using carboplatin, doxorubicin and gemcitabine are indicated for bitches with mammary neoplasms and can be used alone or in combination with cyclophosphamide and COX-2 inhibitors (Lavalle et al. 2012; Cassali et al. 2020). Suryawanshi (2021) concluded, in their study, that surgical excision combined with chemotherapy with cyclophosphamide is an effective protocol for the treatment of malignant mammary tumours in dogs, with minimal toxicity and it may be possible to increase the quality and survival of patients.

In both human and veterinary medicine, selective COX-2 inhibitors are indicated in the treatment of mammary carcinomas, associated with other antitumour drugs. To obtain good treatment results with COX-2 inhibitors, COX-2 expression in tumours must be previously determined (Kaszak et al. 2018).

Patients with breast cancer should be monitored every two months. In the first six months with clinical evaluations, chest X-rays in three positions and an abdominal ultrasound; subsequently, such exams must be carried out every three months, for two years (Cassali et al. 2020).

### Inflammatory carcinoma

Inflammatory mammary carcinoma (IMC) is a highly aggressive subtype of mammary gland tumour that occurs spontaneously in women and bitches (Pena et al. 2003; Cassali et al. 2014), characterised as a progressive disease with a high mortality rate (Marconato et al. 2009).

Its clinical presentation is like mastitis or dermatitis, a hardened plaque with an appearance that resembles an orange peel, presenting extensive inflammation of the skin overlying the mammary glands, oedema and pain involving the axillary, mammary and inguinal regions. Patients with IMC may present with systemic signs of generalised weakness, anorexia and metastasis (Perez Alenza et al. 2001).

It is characterised by the presence of any subtype of carcinoma associated or not with an intense inflammatory reaction, infiltration in the dermis and its superficial lymphatic vessels, with lymphatic tumour emboli, observed on histopathology (Marconato et al. 2009; Cassali et al. 2014). It is noteworthy that for a correct diagnosis of IMC, the histopathological evaluation must be associated with the observation of inflammatory clinical signs (pain, heat, oedema, redness) (Goldschmidt et al. 2011).

Although the recommended treatment for breast carcinomas is surgical excision, in the case of IMC, it is difficult to define the surgical margins due to the extensive inflammation. In this case, chemotherapy and/or palliative treatments are preferable (Raposo et al. 2017).

There is no consensus on the best chemotherapy protocol to be used in dogs with IMC (Raposo et al. 2017), but clinical studies have shown a satisfactory response in relation to the prognosis of dogs treated with COX-2 inhibitors (piroxicam, firocoxib), alone or in combination with other chemotherapy drugs such as doxorubicin, carboplatin, cyclophosphamide and mitoxantrone (Clemente et al. 2009; Marconato et al. 2009; Gregorio et al. 2013). Carprofen at a dose of 4.4 mg/kg, orally, every 24 h and firocoxib at a dose of 5 mg/kg, orally, every 24 h, also provide clinical improvement in these patients (De Nardi et al. 2016).

This carcinoma has a poor prognosis and the average survival time varies from weeks to a few months (Marconato et al. 2009). According to Raposo et al. (2017), prognostic studies in dogs with IMC are challenging, because most of these animals will be euthanised, given the severity of the clinical signs presented, rather than dying from advanced metastatic disease.

### Prevention

The role of sex hormones in the development of mammary tumours in bitches is supported by their high incidence in unspayed bitches or when ovariohysterectomy (OH) is performed after the second oestrous cycle (Lana et al. 2007; Kamiguchi et al. 2016).

Due to this, early castration (before the first oestrus) is recognised as the main measure for preventing mammary neoplasms in bitches (Lana et al. 2007; Kamiguchi et al. 2016), reinforcing that hormonal exposure during life increases the predisposition to developing mammary tumours.

According to Misdorp (2002), young bitches castrated before the first oestrus have a 0.5% risk of developing mammary cancer, while those that underwent the castration procedure after the first oestrous cycle have an 8% risk, and those that underwent castration after the second oestrus have a 26% greater risk of developing the neoplasm. OH performed after two and a half years of age, is not prophylactic, as it is no longer possible to inhibit hormonal action (Withrow et al. 2014).

Some research has reported adverse effects of OH before the first oestrus, such as urinary incontinence and increased risk of other types of neoplasms, such as lymphoma, mast cell tumour and osteosarcoma (Riva et al. 2013; Hart et al. 2020a; Cooley et al. 2002).

Cooley et al. (2002) reported that Rottweiler bitches, castrated before one year of age, had a three to four times higher risk of developing osteosarcoma when compared to non-castrated bitches.

However, Riva et al. (2013) observed that when castration in bitches is carried out after 12 months of age, the risk of developing hemangiosarcoma is four times greater when subjected to early castration or those not castrated.

In a recent study carried out by Hart et al. (2020b), with mixed-breed dogs, the authors assessed the risk of developing orthopaedic problems and neoplasms such as hemangiosarcoma, mast cell tumour, lymphoma and osteosarcoma according to the animal’s reproductive status and weight. These researchers did not observe an increase in the incidence of cancer related to early castration, however, dogs over 20 kilograms castrated before one year of age showed a high incidence of orthopaedic conditions, suggesting the need for a personalised surgical castration approach, according to the size of the animals.

However, in another study, this same group of researchers, observed that Shih-Tzu bitches castrated between six and 11 months increased the risk of developing neoplasms (Hart et al. 2020a).

For Cassali et al. (2020), more studies are still needed to determine the best time to perform castration. These researchers suggested that, when the main objective of castration is the prevention of mammary neoplasia, it should be carried out between the first and second oestrus. However, specific characteristics of each breed must be considered when making a decision.

## FINAL CONSIDERATIONS

Mammary neoplasms in bitches are frequently diagnosed in clinical surgical care for small animals. In this way, knowledge about the main aspects of this tumour, its diagnosis, as well as therapeutic and prophylaxis options become fundamental for the success of treatment and maintenance of the patient’s quality of life.

## References

[R1] Andrade FH, Figueiroa FC, Bersano PR, Bissacot DZ, Rocha NS. Malignant mammary tumor in female dogs: Environmental contaminants. Diagn Pathol. 2010 Jun 30;5:45. 20587072 10.1186/1746-1596-5-45 PMC2909155

[R2] de Araujo MR, Campos LC, Ferreira E, Cassali GD. Quantitation of the regional lymph node metastatic burden and prognosis in malignant mammary tumors of dogs. J Vet Intern Med. 2015 Sep-Oct;29(5):1360-7. 26130166 10.1111/jvim.13576 PMC4858035

[R3] Bartels KE, Ferguson HR, Gillete EL, Ferguson HL. Simultaneous bilateral mastectomy in the dog. Vet Surg. 1978;7(4):97-102.

[R4] Batschinski K, Tedardi MV. Estadiamento clinico das neoplasias [Clinical staging of neoplasmas]. In: Daleck CR, De Nardi AB. Oncologia em caes e gatos [Oncology in dogs and cats]. 2^nd^ ed. Rio de Janeiro: Roca; 2016. 98 p. Portuguese.

[R5] Beauvais W, Cardwell JM, Brodbelt DC. The effect of neutering on the risk of mammary tumours in dogs – A systematic review. J Small Anim Pract. 2012 Jun;53(6):314-22. 22647210 10.1111/j.1748-5827.2011.01220.x

[R6] Beer P, Chiti LE, Nolff MC. The role of sentinel node mapping and lymphadenectomies in veterinary surgical oncology. Lymph. 2023 Jan 10;1(1):2-18.

[R7] Belotta AF, Gomes MC, Rocha NS, Melchert A, Giuffrida R, Silva JP, Mamprim MJ. Sonography and sonoelastography in the detection of malignancy in superficial lymph nodes of dogs. J Vet Intern Med. 2019 May;33(3):1403-13. 30883935 10.1111/jvim.15469 PMC6524127

[R8] Beserra HEO, Greandi F, Dufloth RM, Pinheiro LGP, Miot HA, Vexenat SCOR, Rocha NS. Metastasis of mammary carcinoma in bitches: Evaluation of the sentinela lymph node technique. Adv Breast Canc Res. 2016 Jan;5(2):58-65.

[R9] Bianchi SP, Gomes C, Pavarini SP, Mombach VS, Santos FR, Vieira LC, Oliveira LO, Contesini EA. Linfonodo axilar como sentinela de neoplasia mamaria em cadelas [Axillary lymph node as sentinel of mammary neoplasia in bitch]. Pesq Vet Bras. 2018 Apr;38(4):692-95. Portuguese.

[R10] Biondi LR, Gentile LB, Rego AAMS, Noronha NP, Dagli MLZ. Canine mammary tumors in Santos, Brazil: Clinicopathological and survival profile. Braz J Vet Res Anim Sci. 2014;51(3):252-62.

[R11] Boston S, Henderson RA Jr. Role of surgery in multimodal cancer therapy for small animals. Vet Clin North Am Small Anim Pract. 2014 Sep;44(5):855-70. 25174903 10.1016/j.cvsm.2014.05.008

[R12] Brissot HN, Edery EG. Use of indirect lymphography to identify sentinel lymph node in dogs: A pilot study in 30 tumours. Vet Comp Oncol. 2017 Sep;15(3):740-53. 26899244 10.1111/vco.12214

[R13] Buitrago F, Uemura G, Sena MCF. Fatores prognosticos em cancer de mama. Comunicacao em Ciencia da Saude [Prognostic factors in breast cancer. Communication in health science]. 2011;22:69-82. Portuguese.

[R14] Carvalho MI, Pires I, Prada J, Raposo TP, Gregorio H, Lobo L, Queiroga FL. High COX-2 expression is associated with increased angiogenesis, proliferation and tumoural inflammatory infiltrate in canine malignant mammary tumours: A multivariate survival study. Vet Comp Oncol. 2017 Jun;15(2):619-31. 26792550 10.1111/vco.12206

[R15] Cassali GD, Lavalle GE, De Nardi AB, Ferreira E, Bertagnolli AC, Estrela-Lima A, Alessi AC, Daleck CR, Salgado BS, Fernandes CG, Sobral RA, Amorim RL, Gamba CO, Damasceno KA, Auler PA, Magalhaes GM, Silva JO, Raposo JB, Ferreira AMR, Oliveira LO, Malm C, Zuccari DAPC, Tanaka NM, Ribeira LR, Campos LC, Souza CM, Leite JS, Soares LMC, Cavalcanti MF, Fonteneles ZGC, Schuch ID, Paniago J, Oliveira TS, Terra EM, Castanheira TLL, Feliz AOC, Carvalho GD, Guim TN, Garrido E, Fernandes SC, Maia FCL, Dagli MLZ, Rocha NS, Fukumasu H, Grandi F, Machado JP, Silva SMMS, Bezerril JE, Frehse MS, Paes de Almeida EC, Campos CB. Consensus for the diagnosis, prognosis and treatment of canine mammary tumours. Braz J Vet Pathol. 2011;4(2):153-80.

[R16] Cassali GD, Lavalle GE, Ferreira E, Estrela-Lima A, De Nardi AB, Ghever C, Sobral RA, Amorim RL, Oliveira LO, Sueiro FAR, Beserra HEO, Bertagnolli AC, Gamba CO, Damasceno KA, Campos CB, Araujo MR, Campos LC, Monteiro LN, Nunes FC, Horta RS, Reis DC, Luvizotto MCR, Magalhaes GM, Raposo JB, Ferreira AMR, Tanaka NM, Grandi F, Ubukata R, Batschinski K, Terra EM, Salvador RCL, Jark PC, Delecrodi JER, Nascimento NA, Silva DN, Silva LP, Ferreira KCRS, Frehse MS, Di Santis GW, Silva EO, Guim TN, Kerr B, Cintra PP, Silva FBF, Leite JS, Mello MFV, Ferreira MLG, Fukumasu H, Salgado BS, Torres R. Consensus for the diagnosis, prognosis and treatment of canine mammary tumors – 2013. Braz J Vet Pathol. 2014;7(2):38-69.

[R17] Cassali GD, Jark PC, Gamba C, Damasceno KA, Estrela-Lima A, De Nardi AB, Ferreira E, Horta RS, Firmo BF, Sueiro FAR, Rodrigues LCS, Nakagak KYR. Consensus regarding the diagnosis, prognosis and treatment of canine and feline mammary tumors – 2019. Braz J Vet Pathol. 2020;13(3):555-74.

[R18] Cavalcanti MF, Cassali GD. Fatores prognosticos no diagnostico clinico e histopatologico dos tumores de mamas em cadelas – Revisao [Prognostic factors in the clinical and histopathological diagnosis of mammary tumors in dogs – Review]. Rev Clin Vet. 2006;11(61):56-63. Portuguese.

[R19] Chandler M, Cunningham S, Lund EM, Khanna C, Naramore R, Patel A, Day MJ. Obesity and associated comorbidities in people and companion animals: A one health perspective. J Comp Pathol. 2017 May;156(4):296-309. 28460795 10.1016/j.jcpa.2017.03.006

[R20] Cleary MP, Grossmann ME, Ray A. Effect of obesity on breast cancer development. Vet Pathol. 2010 Mar;47(2):202-13. 20124008 10.1177/0300985809357753

[R21] Clemente M, De Andres PJ, Pena L, Perez-Alenza MD. Survival time of dogs with inflammatory mammary cancer treated with palliative therapy alone or palliative therapy plus chemotherapy. Vet Rec. 2009 Jul 18;165(3):78-81. 19617612 10.1136/vetrec.165.3.78

[R22] Collivignarelli F, Tamburro R, Aste G, Falerno I, Del Signore F, Simeoni F, Patsikas M, Gianfelici J, Terragni R, Attorri V, Carluccio A, Vignoli M. Lymphatic drainage mapping with indirect lymphography for canine mammary tumors. Animals (Basel). 2021 Apr 13;11(4):1115. 33924625 10.3390/ani11041115 PMC8070006

[R23] Cooley DM, Beranek BC, Schlittler DL, Glickman NW, Glickman LT, Waters DJ. Endogenous gonadal hormone exposure and bone sarcoma risk. Cancer Epidemiol Biomarkers Prev. 2002 Nov;11(11):1434-40. 12433723

[R24] Cotran RS, Kumar V, Robbins SL. Patologia estrutural e funcional [Structural and functional pathology]. 6^th^ ed. Rio de Janeiro: Guanabara Koogan; 2000. 1400 p. Portuguese.

[R25] De Nardi AB, Daleck CR, Amorin RL, Huppes RR, Uscategui RAR, Rodaski S, Calderon C, Neto RT. Expresion de la ciclooxigenasa-2 en los carcinomas mamarios caninos primarios metastasicos y no metastasicos [Cyclooxigenase-2 expression in primary metastatic and non metastatic canine mammarian carcinomas]. Arch Med Vet. 2013;45(3):311-6. Spanish.

[R26] De Nardi AB, Ferreira TMMR, Assuncao KA. Neoplasias mamarias [Mammary neoplasms]. In: Daleck CR, De Nardi AB, editors. Oncologia em caes e gatos [Oncology in dogs and cats]. 2^nd^ ed. Rio de Janeiro: Roca; 2016. 730 p. Portuguese.

[R27] de Oliveira Gamba C, Scaratti D, de Andrade VP, Estrela-Lima A, Ferreira E, Cassali GD. Invasive micropapillary carcinoma of the mammary gland in humans and canines: Clinicopathological, immunophenotypical and survival approaches. Res Vet Sci. 2017 Dec;115:189-94. 28475997 10.1016/j.rvsc.2017.04.012

[R28] Dore M, Lanthier I, Sirois J. Cyclooxygenase-2 expression in canine mammary tumors. Vet Pathol. 2003 Mar;40(2):207-12. 12637762 10.1354/vp.40-2-207

[R29] Dobson JM, Lascelles BDX. BSAVA manual of canine and feline oncology. Quedgeley, Gloucester: British Small Animal Veterinary Association; 2011. 364 p.

[R30] Dyce KM, Sack WO, Wensing CJG. Tratado de anatomia veterinaria [Treated of veterinary anatomy]. 4^th^ ed. Rio de Janeiro: Elsevier; 2010. 813 p. Portuguese.

[R31] Estrela-Lima A, Araujo MS, Costa-Neto JM, Teixeira-Carvalho A, Barrouin-Melo SM, Cardoso SV, Martins-Filho OA, Serakides R, Cassali GD. Immunophenotypic features of tumor infiltrating lymphocytes from mammary carcinomas in female dogs associated with prognostic factors and survival rates. BMC Cancer. 2010 Jun 4;10:256. 20525350 10.1186/1471-2407-10-256 PMC2894795

[R32] Ferreira E, Bertagnolli AC, Cavalcanti MF, Schmitt FC, Cassali GD. The relationship between tumour size and expression of prognostic markers in benign and malignant canine mammary tumours. Vet Comp Oncol. 2009 Dec;7(4):230-5. 19891693 10.1111/j.1476-5829.2009.00193.x

[R33] Getty R. Anatomia dos animais domesticos [Anatomy of domestics animals]. 5^th^ ed. Rio de Janeiro: Guanabara Koogan; 2008. 2052 p. Portuguese.

[R34] Giuliano AE, Hunt KK, Ballman KV, Beitsch PD, Whitworth PW, Blumencranz PW, Leitch AM, Saha S, McCall LM, Morrow M. Axillary dissection vs no axillary dissection in women with invasive breast cancer and sentinel node metastasis: A randomized clinical trial. JAMA. 2011 Feb 9;305(6):569-75. 21304082 10.1001/jama.2011.90 PMC5389857

[R35] Goldschmidt M, Pena L, Rasotto R, Zappulli V. Classification and grading of canine mammary tumors. Vet Pathol. 2011 Jan;48(1):117-31. 21266722 10.1177/0300985810393258

[R36] Goldschmidt M, Pena L, Zappulli V. Tumours of the mammary gland. In: Meuton DJ, editor. Tumours in domestic animals. 5^th^ ed. Ames, Iowa: John Wiley & Sons; 2017. p. 723-65.

[R37] Gregorio H, Prada J, Queiroga FL, Lobo L. Use of Firocoxib alone or in combination with metronomic Chlorambucil doses in macroscopic unresectable cancers in dogs. In: Proceedings of the European Society of Veterinary Oncology annual meeting; May 30^th^ – Jun 1^st^ 2013; Lisbon; 2013. p. 62.

[R38] Guimaraes MJ, Carvalho MI, Pires I, Prada J, Gil AG, Lopes C, Queiroga FL. Concurrent expression of cyclo-oxygenase-2 and epidermal growth factor receptor in canine malignant mammary tumours. J Comp Pathol. 2014 Jan;150(1):27-34. 24060154 10.1016/j.jcpa.2013.07.005

[R39] Hart BL, Hart LA, Thigpen AP, Willits NH. Assisting decision-making on age of neutering for mixed breed dogs of five weight categories: Associated joint disorders and cancers. Front Vet Sci. 2020a Jul 31;7:472. 32851043 10.3389/fvets.2020.00472 PMC7412743

[R40] Hart BL, Hart LA, Thigpen AP, Willits NH. Assisting decision-making on age of neutering for 35 breeds of dogs: Associated joint disorders, cancers, and urinary incontinence. Front Vet Sci. 2020b Jul 7;7:388. 32733924 10.3389/fvets.2020.00388 PMC7359819

[R41] Henderson BE, Feigelson HS. Hormonal carcinogenesis. Carcinogenesis. 2000 Mar;21(3):427-33. 10688862 10.1093/carcin/21.3.427

[R42] Horta RS, Costa MP, Lavalle RB, Cassali GD. Fatores prognosticos e preditivos dos tumores caninos definidos com o auxilio da imuno-histoquimica [Prognostic and predictive factors of canine tumours defined with immunohistochemistry‘s assistance]. Cienc Rural. 2012 Jun;42(6):1033-9. Portuguese.

[R43] Horta RS, Lavall GE, Castro Cunha RM, Moura LL, Araujo RB, Cassali GD. Influence of surgical technique on overall survival, disease free interval and new lesion development interval in dogs with mammary tumors. Adv Breast Canc Res. 2014 Apr;3(2):38-46.

[R44] Horta RS, Figueiredo MS, Lavalle GE, Costa MP, Cunha RM, Araujo RB. Surgical stress and postoperative complications related to regional and radical mastectomy in dogs. Acta Vet Scand. 2015 Jun 24;57(1):34. 26104069 10.1186/s13028-015-0121-3 PMC4480898

[R45] Humphrey LL, Helfand M, Chan BK, Woolf SH. Breast cancer screening: A summary of the evidence for the U.S. Preventive Services Task Force. Ann Intern Med. 2002 Sep 3;137(5 Part 1):347-60. 12204020 10.7326/0003-4819-137-5_part_1-200209030-00012

[R46] Kamiguchi IE, Moreira IM, Silva Trindade F, Zahn A, Sousa Rocha N. Mammary neoplasms in female dogs: Identification of cytopathological criteria for malignancy. J Cytol Histol. 2016;7(1):1-5.

[R47] Karayannopoulou M, Lafioniatis S. Recent advances on canine mammary cancer chemotherapy: A review of studies from 2000 to date. Rev Med Vet. 2016 Aug;167(7/8):192-200.

[R48] Kaszak I, Ruszczak A, Kanafa S, Kacprzak K, Krol M, Jurka P. Current biomarkers of canine mammary tumors. Acta Vet Scand. 2018 Oct 29;60(1):66. 30373614 10.1186/s13028-018-0417-1 PMC6206704

[R49] Kim NH, Lim HY, Im KS, Shin JI, Kim HW, Sur JH. Evaluation of clinicopathological characteristics and oestrogen receptor gene expression in oestrogen receptor-negative, progesterone receptor-positive canine mammary carcinomas. J Comp Pathol. 2014 Jul;151(1):42-50. 24913515 10.1016/j.jcpa.2014.04.001

[R50] Kristiansen VM, Pena L, Diez Cordova L, Illera JC, Skjerve E, Breen AM, Cofone MA, Langeland M, Teige J, Goldschmidt M, Sorenmo KU. Effect of ovariohysterectomy at the time of tumor removal in dogs with mammary carcinomas: A randomized controlled trial. J Vet Intern Med. 2016 Jan-Feb;30(1):230-41. 26687731 10.1111/jvim.13812 PMC4913665

[R51] Lana SE, Rutteman GR, Withrow SJ. Tumors of the mammary gland. In: Withrow SJ, Vail DM, editors. Withrow and MacEwen’s Small animal clinical oncology. 4^th^ ed. St. Louis: Saunders Elsevier; 2007. p. 619-38.

[R52] Lavalle GE, Bertagnolli AC, Tavares WL, Cassali GD. Cox-2 expression in canine mammary carcinomas: Correlation with angiogenesis and overall survival. Vet Pathol. 2009 Nov;46(6):1275-80. 19605908 10.1354/vp.08-VP-0226-C-FL

[R53] Lavalle GE, De Campos CB, Bertagnolli AC, Cassali GD. Canine malignant mammary gland neoplasms with advanced clinical staging treated with carboplatin and cyclooxygenase inhibitors. In Vivo. 2012 May-Jun;26(3):375-9. 22523289

[R54] Lim HY, Im KS, Kim NH, Kim HW, Shin JI, Yhee JY, Sur JH. Effects of obesity and obesity-related molecules on canine mammary gland tumors. Vet Pathol. 2015 Nov;52(6):1045-51. 25883120 10.1177/0300985815579994

[R55] Litterine-Kaufman J, Casale SA, Mouser PJ. Prevalence of malignancy in masses from the mammary gland region of dogs with single or multiple masses. J Am Vet Med Assoc. 2019 Oct 1;255(7):817-20. 31517582 10.2460/javma.255.7.817

[R56] Majeski SA, Steffey MA, Fuller M, Hunt GB, Mayhew PD, Pollard RE. Indirect computed tomographic lymphography for iliosacral lymphatic mapping in a cohort of dogs with anal sac gland adenocarcinoma: Technique description. Vet Radiol Ultrasound. 2017 May;58(3):295-303. 28185349 10.1111/vru.12482

[R57] Marconato L, Romanelli G, Stefanello D, Giacoboni C, Bonfanti U, Bettini G, Finotello R, Verganti S, Valenti P, Ciaramella L, Zini E. Prognostic factors for dogs with mammary inflammatory carcinoma: 43 cases (2003–2008). J Am Vet Med Assoc. 2009 Oct 15;235(8):967-72. 19827983 10.2460/javma.235.8.967

[R58] Martins LR, Lopes MD. Pseudociese canina [Canine pseudociesis]. Rev Bras Reprod Anim. 2005;29(3/4):137-41. Portuguese.

[R59] Matos AJ, Baptista CS, Gartner MF, Rutteman GR. Prognostic studies of canine and feline mammary tumours: The need for standardized procedures. Vet J. 2012 Jul;193(1):24-31. 22296767 10.1016/j.tvjl.2011.12.019

[R60] Maues T, Israel CB, Ferreira MLG, Ferreira AMR. Uso do corante azul de metileno a 2% na localizacao do linfonodo axilar em cadelas (Canis familiaris – Linnaeus, 1758) [Use of 2% methylene blue dye forlocation of axillary lymph node in bitches (Canis familiaris – Linnaeus, 1758)]. Braz J Vet Res Anim Sci. 2016;53(1):32-8. Portuguese.

[R61] Millanta F, Asproni P, Canale A, Citi S, Poli A. COX-2, mPGES-1 and EP2 receptor immunohistochemical expression in canine and feline malignant mammary tumours. Vet Comp Oncol. 2016 Sep;14(3):270-80. 24824420 10.1111/vco.12096

[R62] Misdorp W. Tumors of the mammary gland. In: Meuten DJ, editor. Tumors in domestic animals. Iowa State: University of California; 2002. p. 575-606.

[R63] Monteiro GA, Novaes JR, Carvalho Junior JD, Rio JA, Ribeiro LLS, Silva LP, Silva MV, Pereira NLL, Faria RA, Kerner YG, Neves NMBC. O dilema da decisao da mastectomia bilateral como prevencao do cancer de mama: Aspectos eticos e bioeticos [The dilemma of the decision of bilateral mastectomy as breast cancer preventive procedure: Ethical and bioethical aspects]. Bioethicos. 2011;5(4):443-50. Portuguese.

[R64] Mohr A, Luder Ripoli F, Hammer SC, Willenbrock S, Hewicker-Trautwein M, Kielbowicz Z, Murua Escobar H, Nolte I. Hormone receptor expression analyses in neoplastic and non-neoplastic canine mammary tissue by a bead based multiplex branched DNA assay: A gene expression study in fresh frozen and formalin-fixed, paraffin-embedded samples. PLoS One. 2016 Sep 20;11(9):e0163311. 27649560 10.1371/journal.pone.0163311 PMC5029807

[R65] Moulton JE. Tumors of the mammary gland. In: Moulton JE, editor. Tumors in domestic animals. 3^rd^ ed. Los Angeles: University of California Press; 1990. p. 518-52.

[R66] Nagata WB, Perri SHV, Eugenio FR, Laranjeira MG, Andrade AL. Perfil epidemiologico da neoplasia mamaria canina em Aracatuba: Uma abordagem estatistica [Epidemiological profile of canine mammary neoplasia in Aracatuba: A statistical approach]. Rev Estat (UFOP). 2014;3(3):669-73. Portuguese.

[R67] Novosad CA. Principios de tratamento de tumores da glandula mamaria [Principles of treatment for mammary gland tumors]. Clin Tech Small Anim Pract. 2003 May;18(2):107-9. Portuguese. 12831071 10.1053/svms.2003.36625

[R68] Nowak M, Madej JA, Pula B, Dziegiel P, Ciaputa R. Expression of matrix metalloproteinase 2 (MMP-2), E-cadherin and Ki-67 in metastatic and non-metastatic canine mammary carcinomas. Ir Vet J. 2016 Aug 2;69:9. 27486511 10.1186/s13620-016-0068-3 PMC4969974

[R69] Nunes FC, Gamba CO, Damasceno KA, Campos CB, Horta RS, Araujo MR, Monteiro LN, Lavalle GE, Ferreira E, Cassali GD. Analysis of clinico-pathological data, therapeutical conduct and overall survival of canine mammary lesions attended at the veterinary hospital of the Federal University of Minas Gerais (UFMG). Braz J Vet Pathol. 2014 Jul;7(2):122-6.

[R70] Nunes FC, Campos CB, Teixeira SV, Bertagnolli AC, Lavalle GE, Cassali GD. Epidemiological, clinical and pathological evaluation of overall survival in canines with mammary neoplasms. Arq Bras Med Vet Zootec. 2018 Nov-Dec;70(6):1714-22.

[R71] Nunes FC, Damasceno KA, de Campos CB, Bertagnolli AC, Lavalle GE, Cassali GD. Mixed tumors of the canine mammary glands: Evaluation of prognostic factors, treatment, and overall survival. Vet Anim Sci. 2019 Sep 22;7:100039. Erratum in: Vet Anim Sci. 2020 Mar 21;9:100109. Erratum in: Vet Anim Sci. 2020 Dec 9;11:100156. 32734062 10.1016/j.vas.2018.09.003 PMC7386670

[R72] Oliveira Filho JC, Kommers GD, Masuda EK, Marques BMFPP, Fighera RA, Irigoyen LF, Barros SL. Estudo retrospectivo de 1 647 tumores mamarios em caes [Retrospective study of 1 647 mammary gland tumors in dogs]. Pesq Vet Bras. 2010;30(2):177-85. Portuguese.

[R73] Oliveira BC, Rosso GS, Sartor MD, Souza FC, Cardoso E. Vantagens do rastreamento precoce de metastases por tomografia computadorizada na rotina clinica oncologica de tumores mamarios em cadelas: Revisao de literatura [Advantages of early metastasis screening by computed tomography in the clinical oncology routine of breast tumors in bitches: Literature review]. Rev Educ Contin Med Vet Zootec. CRMV-SP. 2021;19(1):1-13. Portuguese.

[R74] Papazoglou LG, Basdani E, Rabidi S, Patsikas MN, Karagiannopolou M. Current surgical options for mammary tumor removal in dogs. J Veter Sci Med. 2014 Feb;2(1):1-5.

[R75] Paulinelli RR, Freitas-Junior R, Rahal RM, Oliveira LF, Vilela MH, Moreira MA, Alves KL, Peleja MB, Resende TC. A prospective randomized trial comparing patent blue and methylene blue for the detection of the sentinel lymph node in breast cancer patients. Rev Assoc Med Bras (1992). 2017 Feb;63(2):118-23. 28355372 10.1590/1806-9282.63.02.118

[R76] Paz WA, Paim SP, Mello GL. Biopsia de linfonodo sentinela: Experiencia clinica [Sentinel lymph node biopsy: Clinical experience]. Rev Bras Cancerol. 2001 Sep;47(3):309-15. Portuguese.

[R77] Pena L, Silvan G, Perez-Alenza MD, Nieto A, Illera JC. Steroid hormone profile of canine inflammatory mammary carcinoma: A preliminary study. J Steroid Biochem Mol Biol. 2003 Feb;84(2-3):211-6. 12711005 10.1016/s0960-0760(03)00030-x

[R78] Pereira CT, Luiz Navarro Marques F, Williams J, Wlademir De Martin B, Primo Bombonato P. 99mTc-labeled dextran for mammary lymphoscintigraphy in dogs. Vet Radiol Ultrasound. 2008 Sep-Oct;49(5):487-91. 18833961 10.1111/j.1740-8261.2008.00414.x

[R79] Perez Alenza MD, Tabanera E, Pena L. Inflammatory mammary carcinoma in dogs: 33 cases (1995–1999). J Am Vet Med Assoc. 2001 Oct 15;219(8):1110-4. 11700710 10.2460/javma.2001.219.1110

[R80] Pinheiro LGP, Moraes MO, Soares AH, Lopes AJT, Naguere MASP, Gondim FAL, Brandao CB, Nascimento DCH, Soares JPH, Meneses e Silva JM. Estudo experimental de linfonodo sentinela na mama da cadela com azul patente e tecnecio Tc99m [Experimental study of the sentinel lymph node in the dog breast using blue dye and technetium Tc99m]. Acta Cir Bras. 2003 Dec;18(6):545-52. Portuguese.

[R81] Queiroga F, Lopes CC. Tumores mamarios caninos: Novas perspectivas [Canine mammary tumors: New perspectives]. In: Congresso de Ciências Veterinárias [Proceedings of the Veterinary Sciences Congress]; 2002 Oct 10-12; Lisboa: Sociedade Portuguesa de Ciências Veterinárias; 2002. p. 183-90. Portuguese.

[R82] Queiroga FL, Pires I, Lobo L, Lopes CS. The role of Cox-2 expression in the prognosis of dogs with malignant mammary tumours. Res Vet Sci. 2010 Jun;88(3):441-5. 19939424 10.1016/j.rvsc.2009.10.009

[R83] Ramos-Vara JA, Kiupel M, Baszler T, Bliven L, Brodersen B, Chelack B, Czub S, Del Piero F, Dial S, Ehrhart EJ, Graham T, Manning L, Paulsen D, Valli VE, West K; American Association of Veterinary Laboratory Diagnosticians Subcommittee on Standardization of Immunohistochemistry. Suggested guidelines for immunohistochemical techniques in veterinary diagnostic laboratories. J Vet Diagn Invest. 2008 Jul;20(4):393-413. 18599844 10.1177/104063870802000401

[R84] Raposo TP, Arias-Pulido H, Chaher N, Fiering SN, Argyle DJ, Prada J, Pires I, Queiroga FL. Comparative aspects of canine and human inflammatory breast cancer. Semin Oncol. 2017 Aug;44(4):288-300. 29526258 10.1053/j.seminoncol.2017.10.012

[R85] Reese S, Budras KD, Mulling C, Bragulla H, Konig HE. Glandula mamaria (mamma, uber, mastos) [Mammary gland (mamma, uber, mastos)]. In: Konig HE, Liebich HG, editors. Anatomia dos animais domesticos [Anatomy of domestics animals]. 4^th^ ed. Porto Alegre: Artmed; 2011. 787 p. Portuguese.

[R86] Reis FR, Barreira APB, Castro V, Castro JLC, Suzano SMC, Rocha A. Indicios sobre a correlacao entre diferentes metodos diagnosticos em casos de tumor de mama em cadelas [Evidence about correlation of breast bitch tumor casesand different methods diagnostic]. Rev Eletro Novo Enfoque. 2010;9(9):14-31. Portuguese.

[R87] Ribas CR, Dornbusch PT, Faria MR, Wouk AFPF, Cirio SM. Alteracoes clinicas relevantes em cadelas com neoplasias mamarias estadiadas [Relevant clinical changes in female dogs with mammary neoplasias staged]. Arch Vet Sci. 2012;17(1):60-8. Portuguese.

[R88] Riva GT, Hart BL, Farver TB, Oberbauer AM, Messam LL, Willits N, Hart LA. Neutering dogs: Effects on joint disorders and cancers in golden retrievers. PLoS One. 2013;8(2):e55937. 23418479 10.1371/journal.pone.0055937 PMC3572183

[R89] Rossi F, Korner M, Suarez J, Carozzi G, Meier VS, Roos M, Rohrer Bley C. Computed tomographic-lymphography as a complementary technique for lymph node staging in dogs with malignant tumors of various sites. Vet Radiol Ultrasound. 2018 Mar;59(2):155-62. 29024279 10.1111/vru.12569

[R90] Rutteman G, Withrow S, MacEwen EG. Tumors of the mammary gland. In: Withrow SJ, MacEwen E, editors. Small animal clinical oncology. 3^rd^ ed. Philadelphia: WB Saunders; 2001. p. 455-77.

[R91] Silva AE, Serakides R, Cassali GD. Carcinogenese hormonal e neoplasias hormonio-dependentes [Hormonal carcinogenesis and hormone-dependent neoplasms]. Cienc Rural. 2004 Mar-Apr;34(2):625-33. Portuguese.

[R92] Sirivaidyapong S. Dogs with mammary gland tumors and the feeding dietary types. In: Proceedings of 28^th^ World Congress of the World Small Animal Veterinary Association; 2003 Oct 24-27; Bangkok, Thailand; 2003.

[R93] Sorenmo KU, Rasotto R, Zappulli V, Goldschmidt MH. Development, anatomy, histology, lymphatic drainage, clinical features, and cell differentiation markers of canine mammary gland neoplasms. Vet Pathol. 2011 Jan;48(1):85-97. 21147765 10.1177/0300985810389480

[R94] Sorenmo KU, Worley DR, Goldschmidt MH. Tumors of the mammary gland. In: Withrow SJ, Vail DM, Page RP, editors. Small animal clinical oncology. 5^th^ ed. Philadelphia: Saunders Company; 2013. p. 538-56.

[R95] Sorenmo K, Worley D, Zappulli V. Tumors of the mammary gland. In: Vail D, Thamm, D, Liptack J, editors. Small animal clinical oncology. 6^th^ ed. St. Louis, MO: Elsevier; 2020. p. 604-25.

[R96] Suryawanshi RV. Assessment of efficacy and toxicity of cyclophosphamide chemotherapy in canines with malignant mammary tumor: A retrospective study. Vet Med Int. 2021 Aug 12;2021:5520603. 34422254 10.1155/2021/5520603 PMC8378984

[R97] Toribio JMML, Estrela-Lima A, Martins Filho EF, Ribeiro LGR, D’Assis MJMH, Teixeira RG, Damasceno KA, Cassali GD, Costa Neto JM. Caracterizacao clinica, diagnostico histopatologico e distribuicao geografica das neoplasias mamarias em cadelas de Salvador, Bahia [Clinical characterization, histopathologic diagnosis and geoprocessing of mammary tumours in bitches from the city of Salvador, Bahia State]. Rev Ceres. 2012 Jul-Aug;59(4):427-33. Portuguese.

[R98] Tuohy JL, Milgram J, Worley DR, Dernell WS. A review of sentinel lymph node evaluation and the need for its incorporation into veterinary oncology. Vet Comp Oncol. 2009 Jun;7(2):81-91. 19453362 10.1111/j.1476-5829.2009.00183.x

[R99] Uva P, Aurisicchio L, Watters J, Loboda A, Kulkarni A, Castle J, Palombo F, Viti V, Mesiti G, Zappulli V, Marconato L, Abramo F, Ciliberto G, Lahm A, La Monica N, de Rinaldis E. Comparative expression pathway analysis of human and canine mammary tumors. BMC Genomics. 2009 Mar 27;10:135. 19327144 10.1186/1471-2164-10-135 PMC2670324

[R100] Valdivia G, Alonso-Diez A, Perez-Alenza D, Pena L. From conventional to precision therapy in canine mammary cancer: A comprehensive review. Front Vet Sci. 2021 Feb 17;8:623800. 33681329 10.3389/fvets.2021.623800 PMC7925635

[R101] Withrow SJ, Page R, Vail, DM. Withrow and MacEwen’s Small animal clinical oncology. St. Louis: Elsevier Health Sciences; 2014. 750 p.

[R102] Worley DR. Incorporation of sentinel lymph node mapping in dogs with mast cell tumours: 20 consecutive procedures. Vet Comp Oncol. 2014 Sep;12(3):215-26. 22958227 10.1111/j.1476-5829.2012.00354.x

[R103] Zatloukal J, Lorenzova J, Tichy F, Necas A, Kecova H, Kohout P. Breed and age as risk factors for canine mammary tumours. Acta Vet Brno. 2005;74:103-9.

